# Multidisciplinary Approach in the Early Detection of Undiagnosed Connective Tissue Diseases in Patients With Interstitial Lung Disease: A Retrospective Cohort Study

**DOI:** 10.3389/fmed.2020.00011

**Published:** 2020-02-18

**Authors:** Claudio Tirelli, Valentina Morandi, Adele Valentini, Claudia La Carrubba, Roberto Dore, Giovanni Zanframundo, Patrizia Morbini, Silvia Grignaschi, Andrea Franconeri, Tiberio Oggionni, Emiliano Marasco, Ludovico De Stefano, Zamir Kadija, Francesca Mariani, Veronica Codullo, Claudia Alpini, Carlo Scirè, Carlomaurizio Montecucco, Federica Meloni, Lorenzo Cavagna

**Affiliations:** ^1^Division of Pneumology, University and IRCCS Policlinico S. Matteo Foundation, Pavia, Italy; ^2^Division of Rheumatology, University and IRCCS Policlinico S. Matteo Foundation, Pavia, Italy; ^3^Institute of Radiology, University and IRCCS Policlinico S. Matteo Foundation, Pavia, Italy; ^4^Radiology Unit, Isituti Clinici Città di Pavia, Pavia, Italy; ^5^Pathology Unit, University and IRCCS Policlinico S. Matteo Foundation, Pavia, Italy; ^6^Rheumatology Department, Hopital Cochin, Paris, France; ^7^Laboratory of Biochemical-Clinical Analyses, IRCCS Policlinico San Matteo Foundation, Pavia, Italy; ^8^Division of Rheumatology, Arcispedale Sant'Anna, Ferrara, Italy

**Keywords:** interstitial lung disease, connective tissue diseases, multidisciplinary team, early diagnosis, rheumatology, pulmonology, radiology

## Abstract

Interstitial lung disease (ILD) encompasses a wide range of parenchymal lung pathologies with different clinical, histological, radiological, and serological features. Follow-up, treatment, and prognosis are strongly influenced by the underlying pathogenesis. Considering that an ILD may complicate the course of any connective tissue disease (CTD) and that CTD's signs are not always easily identifiable, it could be useful to screen every ILD patient for a possible CTD. The recent definition of interstitial pneumonia with autoimmune features is a further confirmation of the close relationship between CTD and ILD. In this context, the multidisciplinary approach is assuming a growing and accepted role in the correct diagnosis and follow-up, to as early as possible define the best therapeutic strategy. However, despite clinical advantages, until now, the pathways of the multidisciplinary approach in ILD patients are largely heterogeneous across different centers and the best strategy to apply is still to be established and validated. Aims of this article are to describe the organization of our multidisciplinary group for ILD, which is mainly focused on the early identification and management of CTD in patients with ILD and to show our results in a 1 year period of observation. We found that 15% of patients referred for ILD had an underlying CTD, 33% had interstitial pneumonia with autoimmune feature, and 52% had ILD without detectable CTD. Furthermore, we demonstrated that the adoption of a standardized strategy consisting of a screening questionnaire, specific laboratory tests, and nailfold videocapillaroscopy in all incident ILD proved useful in making the right diagnosis.

## Introduction

Interstitial lung disease (ILD) includes a heterogeneous group of parenchymal lung pathologies with different clinical, histological, radiological, and serological features (1). To correctly classify ILD is crucial, since follow-up, treatment, and prognosis are strongly dependent on ILD subtype (2, 3). Considering that ILD may complicate the course of any connective tissue disease (CTD) and that signs of CTD are frequently not easy to identify (4–7), an underlying CTD should be ruled out in every ILD, even when the suspect is low or even absent. The recent definition of interstitial pneumonia with autoimmune features (IPAF) is a further confirmation of the close relationship between CTDs and ILD and of how the borders between the rheumatology and pulmonology practices are day by day less defined (8). In a similar context, the multidisciplinary approach is assuming a growing and accepted role, as the discussion of such cases may help to identify the sometime subtle signs or symptoms of CTD in ILD (9–14). However, despite the clinical advantages, the pathways of the multidisciplinary approach in ILD are largely heterogeneous across different centers and countries, and the best strategy to apply is still to be established and validated, as well as the composition of the multidisciplinary team (i.e., the rheumatologist is not included in many of the described multidisciplinary teams) (15). Furthermore, until now, no screening tools for the early identification of CTD signs and symptoms have been applied in ILD, although previous reports in other settings showed their potential usefulness (16). The inclusion of the rheumatology assessment is an added value for patients (9, 17, 18), and the possibility to start the multidisciplinary pathway from a screening tool seems to be effective in terms of health-care resources optimization. Despite these observations, the best strategy to apply in the multidisciplinary evaluation still has to be defined and validated (19). In this article, we want to describe the organization, and share the first results, of our Multidisciplinary Group for Interstitial Lung Disease (GI-ILD), focusing on the early identification of CTDs in ILD patients referring to our clinics.

## Materials and Methods

### The Pavia Multidisciplinary Group for Interstitial Lung Disease

The GI-ILD is a multidisciplinary group first established in 2015 as a shared initiative between the Rheumatology, Pulmonology, and Radiology Divisions of the University and IRCCS Policlinico San Matteo Foundation of Pavia, a tertiary center of referral in the diagnosis and treatment of CTDs, ILD, and rare pulmonary diseases (4, 5, 20–32). The GI-ILD has been first created for the collegial discussion and revision of the most complex or intriguing cases of ILD through a multidisciplinary discussion (MDD). From 2015 to 2018 the selection of cases to be discussed was on individual basis, as every clinician identified independently the patients. To improve the GI-ILD diagnostic performance at the meantime reducing the risk of missed CTDs diagnosis, from 2018, we established a multistep assessment pathway for newly referred (incident) ILD patients in our hospital. Actually, the process of selection is preliminary to MDD, and it is addressed to focus on patients at increased risk of CTDs, to facilitate the admission to our Multidisciplinary Rheumatology–Pulmonology outpatient clinic for the final assessment.

### GI-ILD General Organization

**Figure 1:**
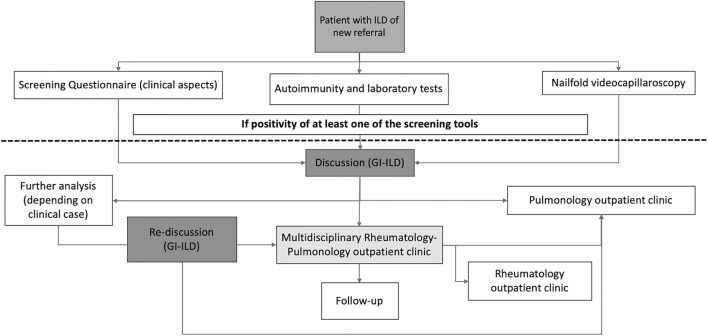
Flow chart of the multidisciplinary discussion we applied in our cohort of newly referring ILD. ILD, interstitial lung disease; GI-ILD, Multidisciplinary Group for Interstitial Lung Disease.

**Figure 2:**
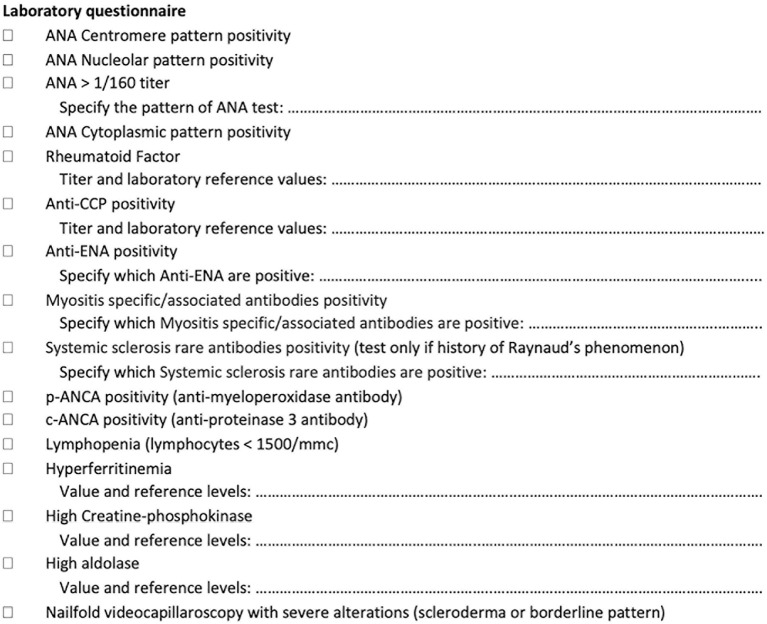
Laboratory tests assessed as a screening tool in newly referring patients with interstitial lung disease.

The organization of the GI-ILD is represented in Figure 1. Our multidisciplinary group includes a team of six Pulmonology, three Rheumatology, two Radiology, and one Pathology specialists supported by their respective fellows. The group's meetings are regularly scheduled every 2 weeks. The GI-ILD is mainly focused on ILD patients first referred to the Pulmonology Unit and without a previous diagnosis of any CTD, to rule out the occurrence of an underlying autoimmune disorder. Patients with a previous diagnosis of CTD have a direct access to the Rheumatology CTD outpatient clinic for diagnosis confirmation. During the first pulmonology assessment, patients are asked to perform or repeat pulmonary function tests (PFT) with diffusion capacity test (DLCO) and to fill in a 12-item questionnaire addressed to identify CTDs features. A previous version of this questionnaire has been applied in another setting with good results (16). When available, all the high-resolution computed tomographies (HRCT) of the chest are evaluated and, if not performed in our center, a copy of the DICOM images are stored for future MDD. Further steps include nailfold videocapillaroscopy (NVC), which is performed independently of Raynaud's Phenomenon (RP) occurrence (25), and a locally established autoimmune and laboratory panel of tests (Figure 2). To avoid possible selection bias, NVC and laboratory tests are, respectively, performed in the Rheumatology and in the Laboratory Division of the IRCCS Policlinico S. Matteo Foundation, a tertiary structure with high skills in the analysis of autoimmune and laboratory tests (33–36). Patients with either a positive questionnaire, NVC, or autoimmune and laboratory panel enter the MDD. During the MDD, the baseline screening results are presented, and the clinical case is discussed, together with the evaluation of chest HRCT images, PFT, and DLCO results. At the end of the discussion, patients without the suspect of an underlying CTD are planned for the regular pulmonology follow-up and treatment according to the suspected or established diagnosis. In case of CTD/IPAF, the patients are referred to the Multidisciplinary Rheumatology–Pulmonology outpatient clinic (RP-OC) for the final diagnostic steps, treatment, and follow-up definition. According to guidelines or expert recommendations, every patient is treated following the best therapeutic option established for the specific diagnosis.

#### First Step

##### Baseline screening questionnaire

The baseline screening questionnaire consists of 12 questions, focusing on 11 CTD manifestations such as RP (question 1), mechanic's hands and pitting scars (question 2), cutaneous sclerosis or puffy fingers (question 3), skin lesions such as heliotrope rash, Gottron's papules, malar rash (question 4), arthritis/inflammatory arthralgias (questions 5 and 6), dry eyes and dry mouth (question 7), oral ulcers (question 8), dysphagia (question 9), proximal muscle weakness (question 10), cutaneous telangiectasias (question 11), and other CTD (and also vasculitis) features such as deep venous thrombosis, sinusitis, and adult-onset asthma (question 12). As pointed-out, every item explores a single manifestation, except for questions 5 and 6, which should be considered as a single item. The positivity of a single item of the baseline questionnaire is sufficient to enter the MDD.

##### Autoimmune and laboratory tests

Laboratory tests (Figure 2) include the antinuclear antibody (ANA) test (for both classic and cytoplasmic positivity) (HEp-2000® Immunoconcepts), an extractable nuclear antigen screen test (EliA SymphonyS; Phadia 250), rheumatoid factor (Rheumatoid factor Flex reagent cartridge Dimension Vista; Siemens), anticyclic citrullinated peptide antibodies (EliA CCP; Phadia 250), antineutrophil cytoplasmic antibodies (ANCA) tests (EliA PR3 S and EliA MPO S: Phadia 250), creatine-phosphokinase, aldolase, erythrocyte sedimentation rate and C-reactive protein, and myositis-specific/myositis-associated antibodies (anti-Jo1, anti-PL7, anti-PL12, anti-OJ, anti-EJ, anti-Pm-Scl 75 and 100, anti-SRP, anti-Mi2, anti-MDA5, anti-NXP2, anti-TIF1gamma, anti-Ku, and anti-Ro52) (EUROLINE, Autoimmune Inflammatory Myopathies 16 Ag; EUROIMMUN). Systemic sclerosis rare antibodies (e.g., anti-PDGFR, anti-Ku, anti-Th/T0, anti-NOR90, anti-fibrillarin, anti-RNA polymerase I and III) [EUROLINE: Systemic Sclerosis (Nucleoli) Profile; Immunoblot EUROIMMUN] are tested only in patients with RP and after the negative result of myositis-specific/myositis-associated antibodies. As a reference value for autoimmune tests, we used the IPAF criteria (8), although for ANA without the nucleolar and anticentromere positivity, we considered as significant every pattern with titers higher than 1/160. Among the positive laboratory findings, we considered also hyperferritinemia and lymphopenia because of some reports as negative prognostic factor in patients with anti-MDA5 syndrome and thus potentially linked to the occurrence of CTD-ILD (37–39). Furthermore, on the basis of previous reports, we included also ANCA antibodies, ANA cytoplasmic positivity, and muscle enzymes assessment (15, 23, 40, 41). In case of a single positive result in autoimmune or laboratory tests, the patient is considered eligible for discussion during the GI-ILD.

##### Nailfold videocapillaroscopy

NVC is performed by the Rheumatology team generally within 10 days from the first pulmonology assessment. A single experienced operator (LC) performs NVC on a VideoCap 13 microscope with 200× magnification. Each exam includes the storage of pictures (three per finger) on a dedicated computer. A second rheumatologist reviews all the stored NVC images and formulates a comment (see Contribution). NVC is systematically performed in all patients according to the consolidated methodology described by Cutolo et al. (42) on each finger of both hands excluding thumbs. Patterns are described as “normal,” “aspecific abnormalities,” and “scleroderma pattern” (25). Scleroderma anomalies include megacapillaries, specific microhemorrhages, neoangiogenesis, or avascular areas (42). Patients with scleroderma anomalies are discussed during the GI-ILD.

#### Second Step

##### Multidisciplinary discussion

The results of the first step are presented during the GI-ILD by the clinician in charge of the patient. HRCT scans are collegially reviewed and discussed, to identify the radiological pattern of lung involvement (43). CT findings are qualitatively analyzed by two radiologists with great expertise on ILD. Similarly, PFTs results are presented, together with other clinically relevant information. In some cases, according to clinical suspicion, further analysis could be asked: muscle magnetic resonance, or muscle biopsy in suspected inflammatory myositis; plan X-rays or Doppler ultrasound of hands and feet in the suspect of arthritis; bronchoscopy with bronchoalveolar lavage fluid examination and cytogram to better characterize alveolitis; and surgical or cryo-biopsies in case of suspected IPF or other forms of fibrosing ILD not otherwise characterizable. Cases for which further analysis are needed enter a rediscussion in the subsequent GI-ILD. After the multidisciplinary discussion, patients diagnosed with a CTD-ILD or IPAF are followed up in the multidisciplinary Rheumatology–Pulmonology outpatient clinic, whereas all the other ILD patients without any rheumatologic involvement continue a regular pulmonology follow-up in a dedicated ILD outpatient clinic. According to the diagnosis, when clinically indicated, specific anti-fibrotic or immunosuppressant therapy is started.

##### Multidisciplinary rheumatology–pulmonology outpatient clinic

The Rheumatology–Pulmonology outpatient clinic is in charge to FMe (Pulmonologist) and to LC (Rheumatologist). At first assessment, patients generally repeat PFT with DLCO. A pulmonology and rheumatology medical examination is then performed, and all the data from the screening phase and of previous tests are reviewed. If a diagnosis is obtained, the appropriate treatment is started according to international guidelines or expert recommendations, and follow-up is planned. PFT + DLCO are repeated every 6 months. Annual HRCT is performed in patients with fibrotic ILD (with or without CTD) or IPF to follow up the stability/progression of fibrotic lung disease, as well as surveillance for possible neoplastic evolution on fibrotic scars or parenchyma. Timing for HRCT follow-up in non-fibrotic CTD-ILD depends largely on clinical and functional aspects. ILD patients diagnosed with established CTDs are subsequently followed in the CTD outpatient clinic and in the Rheumatology–Pulmonology outpatient clinic, while IPAF patients are followed up only in the Rheumatology–Pulmonology outpatient clinic, to identify patients who will develop an established CTD during follow-up. For every definite diagnosis, we adopt well-established classification criteria (8, 44–49), except for the antisynthetase syndrome, because of the lack of shared definitions (8, 50). In fact, in our cohort, every patient testing positive for antisynthetase antibodies is diagnosed with antisynthetase syndrome, in line with our previous reports (5). In case of ILD patients with clinical or laboratory findings suggestive for CTD but without fulfilling any of the existing classification criteria, the final attributed diagnosis is undifferentiated connective tissue disease (45).

##### Data collection

Patient's data from January to December 2018 were collected from electronic health records and medical records of GI-ILD. Every patient signed an informed consent during the first clinical evaluation. The screening questionnaire, autoimmune and laboratory tests, and NVC are collected from patient's medical records, while HRCT and PFT performed at the IRCCS Policlinico S. Matteo Foundation are stored in electronic health records. Copies of outside-performed HRCT DICOM files and PFT are recorded during GI-ILD evaluation and stored locally on a dedicated computer. All patient's medical records are stored in the multidisciplinary Rheumatology–Pulmonology outpatient clinic.

### Statistical Analysis

Patients' characteristics at screening visit have been reported using median and interquartile range for the quantitative variables and absolute/relative frequency values for the qualitative ones. The population study has been divided in three different groups: *connective tissue disease* (CTD), which includes patients diagnosed with established autoimmune rheumatic diseases; *interstitial pneumonia with autoimmune features* (IPAF); and finally, the “other ILD” group, including all the remaining patients. Overall comparison among groups was performed by the one-way ANOVA or by non-parametric Kruskal–Wallis test for quantitative variables and by the chi-square or Fisher's exact test for categorical variables. Statistical significance was set at *p* < 0.05. Significant differences between groups were further evaluated in a *post-hoc* analysis (head-to-head comparison) with a statistical significance set at *p* < 0.025 (Bonferroni correction). Analyses were performed using STATA software package (2018, release 15.1; StataCorp, College Station, TX).

## Results

**Figure 3:**
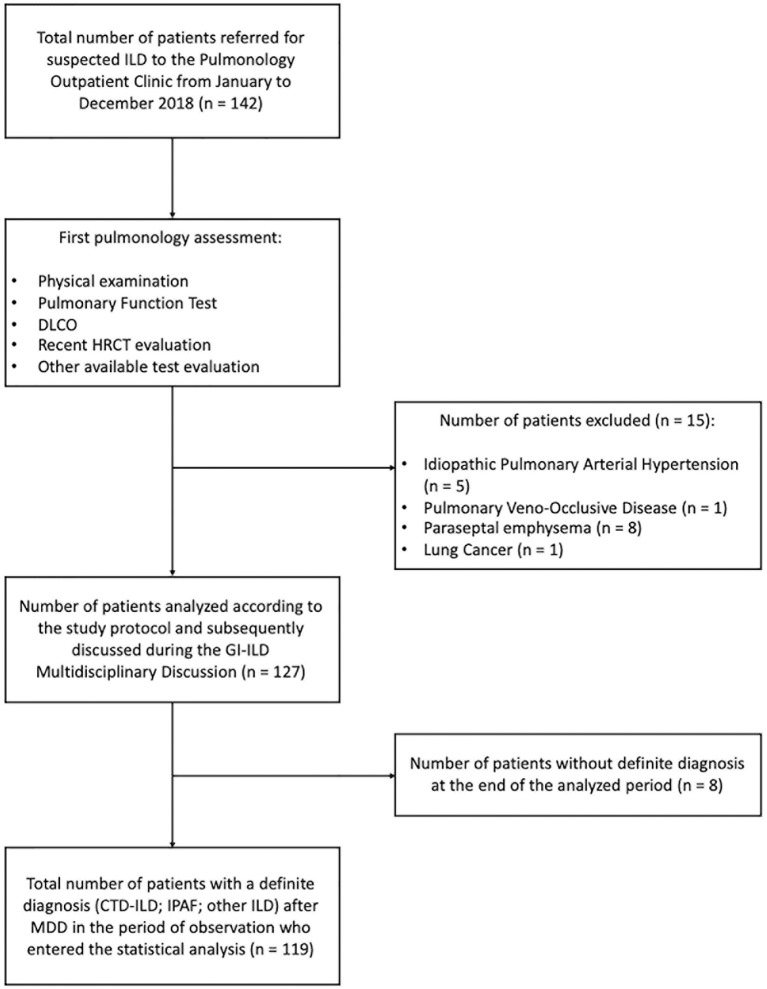
STROBE diagram of the principal selection and analytical phase of the study (STROBE: Strengthening the reporting of observational studies in epidemiology following the EQUATOR network).

We retrospectively analyzed the performance of the GI-ILD group from January to December 2018 (Table 1). A total of 142 patients were referred to the Pulmonology outpatient clinic for a suspected ILD. Fifteen of them were excluded from the multidisciplinary approach after the first screening visit because an alternative diagnosis out of ILD was reached (five idiopathic pulmonary arterial hypertension, one pulmonary veno-occlusive disease; eight chronic obstructive pulmonary disease with paraseptal emphysema mimicking lung cysts or fibrotic air space enlargements; one lung cancer with carcinomatous lymphangitis). Eight patients entered the GI-ILD multidisciplinary discussion, but a definite diagnosis was not yet established at the end of the period considered for the present study, so they were excluded from analysis (STROBE diagram, Figure 3). We thus enrolled 119 patients (59 female and 60 male, 50% each), with a median age at first referral of 70 years (interquartile range, 64–77 years). A CTD was diagnosed in 18 cases (15%: 11 male, 60%; 7 female, 40%) and an IPAF in 39 (33%: 10 male, 26%; 29 female, 74%), together representing 48% of the evaluated cases. The remaining 62 patients (52% of cases: 23 female, 37%; 39 male, 63%) had other forms of ILD (idiopathic, sarcoidosis, exposure related, rare ILD, other origin, i.e., Langerhans cell histiocytosis and lymphangioleiomyomatosis). Sex prevalence was different across the three groups (*p* = 0.036). In a *post-hoc* analysis, we observed that female patients were more commonly classified as IPAF (*p* = 0.010). The age at first referral was not different between patients with (70 years; interquartile range, 64–77) and without CTD/IPAF (70 years; interquartile range, 63–77) (*p* = 0.665). In addition, when considering the referral age of CTD (median, 69 years; interquartile range, 61–73) vs. IPAF (median, 70 years; interquartile range, 64–78 years), we did not find statistically significant differences (*p* = 0.508). The CTD patients were classified as rheumatoid arthritis in four (3%), systemic sclerosis in three (3%), undifferentiated connective tissue disease in three (2%), and antisynthetase syndrome in two (2%) cases, whereas six patients (5%) were classified one each as polymyositis, dermatomyositis, Sjogren syndrome, scleromyositis, amyopathic dermatomyositis, and granulomatosis with polyangiitis. Although granulomatosis with polyangiitis is not a CTD but a vasculitis, we included this patient in the analysis because identified thanks to screening steps. Patients in the “other ILD” group (*n* = 62) were mainly classified as idiopathic pulmonary fibrosis (*n* = 30, 48%). Interestingly, three of these patients (10%) were also diagnosed with polymyalgia rheumatica. The remaining 32 patients were diagnosed as idiopathic non-specific interstitial pneumonia (NSIP) (*n* = 2; 2%), respiratory bronchiolitis–ILD (*n* = 5; 4%), cryptogenic organizing pneumonia (*n* = 2; 2%), lymphoid interstitial pneumonia (*n* = 2; 2%), hypersensitivity pneumonitis (*n* = 5; 4%), secondary organizing pneumonia (OP) (*n* = 3; 2%), postactinic fibrosis (*n* = 1; 1%), sarcoidosis (*n* = 3; 2%), Langerhans cell histiocytosis (*n* = 1; 1%), lymphangioleiomyomatosis (*n* = 1; 1%), combined pulmonary fibrosis and emphysema (*n* = 5; 4%), pleuroparenchymal fibroelastosis (*n* = 2; 2%).

**Table 1:** Results of the GI-ILD multidisciplinary approach in the cohort of patients analyzed (from January to December 2018), see text for details.

**ILD category**		**Specific diagnosis** **(no of patients and %)**		**No ILD patients** **(tot 119)**	**Median Age** **(y) and IQR**	**Male** **(*n* = 60; 50%)**	**Female** **(*n* = 59; 50%)**	**Preliminary screening phase**								**HRCT pattern**				
								**Questionnaire** **(≥1 item pos)**	**Scleroderma pattern** **at NVC**	**Laboratory screening**						**NSIP**	**NSIP + OP**	**UIP** **(def/prob)**	**OP**	**Other** **patterns**
										**ANA**	**Cytoplasmic** **ANA**	**Anti-ENA**	**MSA/MAA**	**RF**	**anti-CCP**					
CTD-ILD		SSc	4 (3%)	18 (15%)	69 (61–73)	11 (57%)	7 (43%)	100%	44%	89%	28%	28%	28%	17%	11%	34%	22%	17%	11%	17%
		RA	3 (3%)																	
		ASSD	2 (2%)																	
		UCTD	3 (2%)																	
		Other CTD	6 (5%)																	
IPAF		IPAF	39 (33%)	39 (33%)	70 (64–78)	10 (26%)	29 (74%)	56%	10%	56%	28%	51%	56%	10%	3%	61%	8%	15%	13%	3%
Other ILD	Idiopathic	IPF	30 (25%)	62 (52%)	70 (63–77)	39 (63%)	23 (37%)	52%	0%	32%	6%	10%	0%	3%	0%	10%	2%	61%	6%	21%
		RB-ILD	5 (4%)																	
		idiopathic NSIP	2 (2%)																	
		idiopathic LIP	2 (2%)																	
		COP	2 (2%)																	
	Sarcoidosis	Sarcoidosis	2 (2%)																	
	Exposure-related	SOP	2 (2%)																	
		Post actinic Fibrosis	1 (1%)																	
	Rare ILD	CPFE	5 (4%)																	
		PPFE	2 (2%)																	
	Myscellanea	HP	5 (4%)																	
		LAM	1 (1%)																	
		LCH	1 (1%)																	
	*p*-value				=0.665	=0.036		<0.001	<0.001	<0.001	=0.007	<0.001	<0.001	=0.791	=0.003	<0.001	=0.008	<0.001	=0.005	=0.035

*ILD, interstitial lung disease; CTD-ILD, connective tissue disease associated ILD; IPAF, interstitial pneumonia with autoimmune features; SSc, systemic sclerosis; RA, rheumatoid arthritis; ASSD, antisynthetase syndrome; UCTD, undifferentiated connective tissue disease; IPF, idiopathic pulmonary fibrosis; RB-ILD, respiratory bronchiolitis-ILD; NSIP, non-specific interstitial pneumonia; LIP, lymphoid interstitial pneumonia; COP, cryptogenic organizing pneumonia; SOP, secondary organizing pneumonia; CPFE, combined pulmonary fibrosis and emphysema; PPFE, pleuroparenchymal fibroelastosis; HP, hypersensitivity pneumonitis; LAM, lymphangioleiomyomatosis; LCH, Langerhans cell histiocytosis. MSA/MAA, myositis specific antibodies/myositis associated antibodies; RF, rheumatoid factor; anti-CCP, anti-cyclic citrullinated peptide antibodies; NVC, nailfold videocapillaroscopy; NSIP, non-specific interstitial pneumonia; NSIP + OP, non-specific interstitial pneumonia + organizing pneumonia; UIP, usual interstitial pneumonia*.

**Figure 4:**
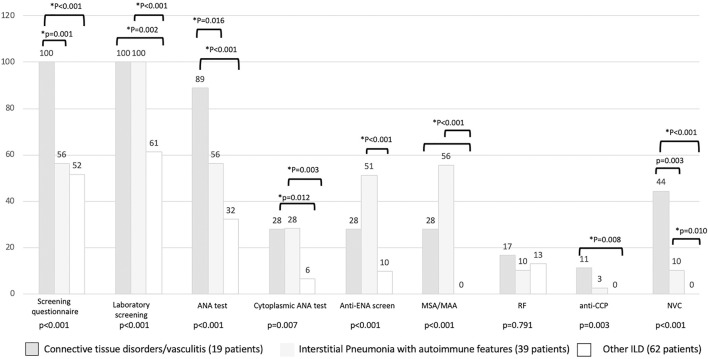
Results (in percentage) of different screening steps according to final patients' classification. *Statistical significance <0.025 for *post-hoc* analysis. MSA/MAA, myositis specific antibodies/myositis associated antibodies; RF, rheumatoid factor; anti-CPP, anti-cyclic citrullinated peptide antibodies; NVC, nailfold videocapillaroscopy.

The results of the first screening step have been reported in Figure 4, stratified according to the diagnosis. The screening questionnaire discriminated well between CTD and other groups (CTD vs. IPAF, *p* = 0.001; CTD vs. other ILD, *p* < 0.001). Laboratory screening was less significantly positive in other ILD (*p* = 0.002 vs. CTD and *p* < 0.001 vs. IPAF). ANA test positivity was more common in CTD group (*p* = 0.016 vs. IPAF and *p* < 0.001 vs. other ILD) and in IPAF group (with respect to other ILD, *p* = 0.016), whereas cytoplasmic positivity of ANA test was more common in CTD and IPAF group with respect to other ILD (*p* = 0.012 and *p* = 0.003, respectively). A similar trend was observed for antiextractable nuclear antigen screen (*p* < 0.001 between IPAF and other ILD) and for myositis-specific and myositis-associated antibodies positivity (for both CTD vs. other ILD and for IPAF vs. other ILD, *p* < 0.001). Rheumatoid factor positivity was not different across the groups (*p* = 0.791), anticyclic citrullinated peptide antibodies were more common in CTD patients with respect to other ILD (*p* = 0.008). Finally, NVC was more frequently positive in CTDs (*p* = 0.003) with respect to IPAF and (*p* < 0.001) with respect to other ILD and in IPAF patients (*p* = 0.010) with respect to other ILD.

**Figure 5:**
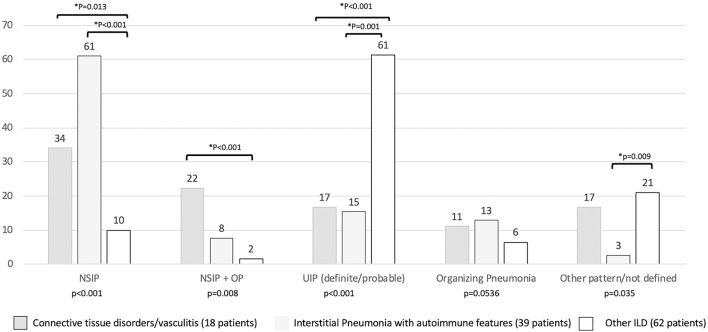
Prevalence (in percentage) of high resolution computed tomography pattern according to final patients' classification. *Statistical significance <0.025 for *post-hoc* analysis. NSIP, non-specific interstitial pneumonia; NSIP + OP, non-specific interstitial pneumonia + organizing pneumonia.

Regarding the HRCT pattern observed (Figure 5), the most prevalent was usual interstitial pneumonia (usual interstitial pneumonia probable, *n* = 47, 44%) followed by NSIP (*n* = 24, 20%), fibrosing NSIP (*n* = 12, 10%) and OP (*n* = 11, 8%). Some patients had superimposed NSIP and OP (*n* = 8, 7%). The distribution of different patterns across the established groups (CTD, IPAF, and other ILD) was statistically different (*p* < 0.001). In particular (Figure 5), NSIP pattern was less common in “other ILD” (*p* = 0.013 vs. CTD and *p* < 0.001 vs. IPAF), the mixed pattern NSIP + OP was more common in CTD than in other ILD (*p* < 0.001), and usual interstitial pneumonia was more common in other ILD (*p* ≤ 0.001 with respect to other groups).

## Discussion

The multidisciplinary collaborative model we applied in the assessment of newly referred ILD seems to be effective in the *de novo* diagnosis of CTD/IPAF. In fact, we correctly classified more than 45% of patients within the spectrum of autoimmune connective tissue disorders. Interestingly, we did not include three patients with polymyalgia rheumatica in the CTD group, although this exclusion could be discussed, in particular if we consider the recently described case series of Sambataro et al. (51).

The results we obtained are relevant, even because our model is reproducible and potentially applicable in other centers after an external validation of the entry questionnaire. The model described seems to improve the overall ILD management, increasing the capability to perform a preliminary differential diagnosis of possible rheumatic disorders underlying an ILD. In fact, the identification of subtle CTD signs is not always easy (52), with the risk to underdiagnose rheumatologic disorders, as we recently showed in a cohort of patients first referring to our hospital with a diagnosis of idiopathic pulmonary arterial hypertension (6). Furthermore, several patients we screened were at the end diagnosed with established CTDs, as a further confirmation that the definition of CTD signs is not rarely troublesome also in ILD patients. The adoption of a self-administered questionnaire seems to represent an added value, allowing the homogeneous evaluation of CTD symptoms in a non-rheumatology setting before the MDD. Moreover, thanks to a well-established collaboration between the Gynecology and the Rheumatology Division of our hospital, a similar approach has been previously applied to a cohort of pregnant women, showing that in patients with positive results, a final diagnosis of CTD was performed in the 25% of cases (16). This is a preliminary confirmation of the potential efficacy of a similar approach in patients referred for ILD, not suspected for but at risk to have a CTD. It is true that continuous clinical exchange within the multidisciplinary team may increase the sensibility of pulmonologist to rheumatology conditions and vice versa, but a standardized preliminary screening for ILD patients may surely reduce the interoperator variability in the assessment of CTD signs. This may be useful, in particular, in smaller secondary centers, were an MDD is not established or feasible. Obviously, as previously suggested, this approach should be validated in other contexts, and support from the National Health Systems and of respective national scientific societies will be necessary for its further application. If the questionnaire is important and generally positive in patients diagnosed with established CTD, in IPAF patients, it is possible to have only laboratory signs of autoimmunity and not clinically relevant features (8). On this basis, during the screening of ILD patients, it is mandatory not only to evaluate the autoimmune profile indicated in the IPAF criteria but also to consider other laboratory tests (15, 23) that have been associated to ILD occurrence, such as the panel we selected. The prototypical example is the cytoplasmic positivity of ANA, which has been linked to the occurrence of antisynthetase syndrome (41). Furthermore, we also enlarged the spectrum of potential rheumatology conditions identified by considering ANCA-associated vasculitis because these conditions are not rarely complicated by the occurrence of ILD (40) and are of primarily interest for both rheumatologists and pulmonologists. One of the patients discussed in the GI-ILD was diagnosed with granulomatosis with polyangiitis, having reported the occurrence of sinusitis together with ANCA positivity at baseline assessment. However, the most useful screening tool we identified was nailfold videocapillaroscopy, which was positive only in case of CTD or IPAF diagnosis, independent to the occurrence of RP, as recently shown in antisynthetase syndrome (25). Although nailfold capillaroscopy should surely enter the routine assessment of every ILD patient, the overall rate of positivity of the test we found in our cohort was quite low.

From the combination of these different domains, during the MDD, we can obtain a series of information that could be helpful in patient's classification, at the same time reducing the number of referral visits before a CTD diagnosis is established. When an ILD occurs, the early identification of CTD or IPAF is crucial and should be carefully considered for the best therapeutic strategy to apply. In fact, an ILD with an autoimmune origin could benefit from immunosuppressant drugs such as cyclophosphamide, cyclosporine, mycophenolate mofetil, azathioprine, and rituximab (20, 53, 54), whereas until now, these patients were simply excluded from the access to anti-fibrotic drugs, such as Nintedanib and Pirfenidone (55). However, the exclusion of these patients from CTD group could be discussed, in particular, if we consider the recently described case series of Sambataro et al. (51) or the promising results of the INBUILD study (56).

In conclusion, with our study, we confirmed that the multidisciplinary approach we applied may be really useful in the identification of CTD-ILD/IPAF in ILD patients without previous rheumatology diagnosis. We suggest that a rheumatologist is necessary in every ILD multidisciplinary team and that, to optimize the diagnostic pathway, a preliminary screening phase with a dedicated questionnaire could be useful. In our opinion, a targeted autoimmune and laboratory profile evaluation and nailfold capillaroscopy should be part of the routine assessment of ILD patients.

## Data Availability Statement

All datasets generated for this study are included in the article/supplementary material.

## Ethics Statement

The GI-ILD is approved and recognized by our Foundation. In line with the Declaration of Helsinki, with our national and institutional regulations, and according to our local Institutional Review Board, we obtained from all patients the signed informed consent for the retrospective use of clinical data collected.

## Author Contributions

TO, RD, AV, FMe, FMa, ZK, PM, and VC organized the GI-ILD. VC, CM, FMe, CS, and LC drafted the screening questionnaire. CA, LC, ZK, CS, FMa, and FMe defined the laboratory test to be searched for in the preliminary phase. LC performed the nailfold videocapillaroscopies, which were reviewed by EM and, in case of conflict, by VC and GZ. AV, RD, and AF performed, analyzed, and discussed CT scans. CT and CL for the pulmonology counterpart. SG, LD, EM, GZ, and VM for the rheumatology counterpart filled the database. LC performed statistical analysis. All Pavia's authors participated to GI-ILD meetings. The paper was mainly drafted by CT, VM, LC, and FMe. CM revised the first draft, and other authors contributed to paper improvement with respect to first version.

### Conflict of Interest

The authors declare that the research was conducted in the absence of any commercial or financial relationships that could be construed as a potential conflict of interest.
